# Gellan Gum Methacryloyl-Based Composite Hydrogels to Promote Murine Primary Macrophage Differentiation

**DOI:** 10.1021/acsomega.5c07273

**Published:** 2025-12-15

**Authors:** Ana Letícia Rodrigues Costa, Jhonatan Rafael de Oliveira Bianchi, Lucimara Gaziola de La Torre, Sang Won Han

**Affiliations:** † Department of Biophysics, Escola Paulista de Medicina, Federal University of Sao Paulo, Sao Paulo, São Paulo 04023-062, Brazil; ‡ School of Chemical Engineering, 28132 State University of Campinas (Unicamp), Campinas, São Paulo 13083-852, Brazil; § Institute of Exact and Technological Sciences, Campus Florestal, Federal University of Viçosa (UFV), Florestal, Minas Gerais 35690-000, Brazil

## Abstract

Injectable hydrogels made from biopolymers present promising platforms for tissue engineering and wound healing applications, particularly because of their tunable mechanical properties and the ability to support cell growth. In this work, we developed and characterized composite hydrogels composed of gellan gum methacryloyl (GMa), unmodified gellan gum (GG), and fibrin (Fib) to investigate their mechanical properties on Raw 264.7 and primary murine macrophages. The mechanical properties and porosity of the hydrogels were tailored by varying the ratio of GGMa and fibrinogen. Hydrogels with higher concentrations of fibrinogen (7–9 mg/mL) exhibited increased stiffness and enhanced porosity and maintained a high cell viability for both lineages. Immunocytochemistry confirmed that primary macrophages preserved their phenotype, expressing crucial markers (F4/80, iNOS, and arginase-1), indicating that the GMa-based hydrogels with optimized fibrinogen concentration can serve as effective scaffolds for macrophage response, promoting M1–M2 macrophage differentiation.

## Graphical abstract

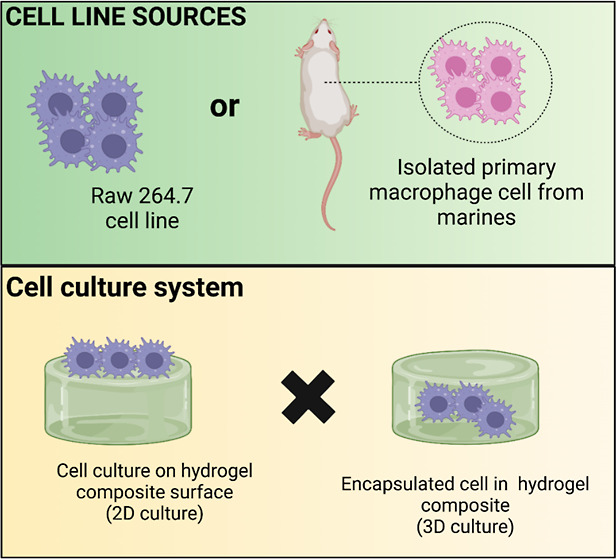

## Introduction

Injectable hydrogels derived from biopolymers have garnered considerable attention as advanced platforms for the targeted delivery of therapeutic biomolecules and cellular components, facilitating disease treatment and accelerating tissue repair processes. Their efficacy is attributed to a sophisticated interplay of biological compatibility, chemical tunability, and distinctive rheological characteristics that enable precise modulation of the cellular microenvironment.^1^ The substantial water content and inherent biocompatibility of these hydrogels underpin robust cellular attachment and expansion, while the engineered chemical milieu—mediated by the presence of RGD motifs or thiol functionalities—further augments cell adhesion and proliferation. Moreover, the tunable rheological characteristics, particularly viscosity, enable precise control over the injectability for site-specific in vivo administration. The controlled biodegradability of these biopolymeric matrices also facilitates the regulated release of encapsulated therapeutic agents.^2,3^ Consequently, these hydrogels possess the capability to encapsulate and facilitate the controlled delivery of a diverse array of biomolecules—including nucleic acids, proteins, growth factors—as well as various cell populations, thereby establishing themselves as advanced platforms for sophisticated applications in tissue engineering.^3,4^

Hydrogels formulated from gellan gum methacryloyl (GMa) exhibit exceptional suitability for advanced cell encapsulation and culture, furnishing a highly hydrated microenvironment that closely emulates the extracellular matrix (ECM). This biomimetic milieu not only facilitates robust cell viability but also actively promotes cellular proliferation, making these hydrogels highly advantageous for sophisticated tissue engineering and regenerative medicine applications.^5–7^ Gellan gum methacryloyl (GMa) is derived from gellan gum (GG), an anionic polysaccharide composed of repeating units of glucose, glucuronic acid, and rhamnose. In the presence of divalent cations such as calcium, GG forms physically cross-linked hydrogel networks. Nevertheless, these ionically cross-linked hydrogels frequently exhibit limited stability owing to dynamic ion-exchange processes.^8^ The methacrylation process mitigates hydrogel instability by covalently incorporating methacrylate moieties through reactions with hydroxyl functionalities. Subsequent UV-induced physical cross-linking of GGMa hydrogels confers superior mechanical integrity, increased rigidity, and a finely tunable degradation profile, thereby advancing their suitability for biomedical applications.^6,9^

GGMa-based hydrogels have shown compatibility for cell culture and encapsulation.^5,10^ One application involves wound healing of injuries; in this case, the hydrogels can deliver growth factors, endothelial cells, and macrophages, promoting angiogenesis and vasculogenesis.^11,12^ To facilitate wound healing through angiogenesis, several biomaterials were utilized to examine endothelial cell proliferation and infiltration.^13–15^ However, vasculature is not the only biological cue that influences the wound healing process. Macrophages are key cells involved in the inflammatory response, differentiating into a spectrum of phenotypes. M1 macrophages represent a proinflammatory phenotype, typically responsible for destroying microorganisms and recruiting other proinflammatory cells to sites of injury. M2 macrophages express factors that promote both pro- and anti-inflammatory responses, aiding in angiogenesis, wound healing, and tissue regeneration.^16^

Accordingly, to mitigate the risk of implant rejection, it is imperative to elucidate the in vivo behavior of biomaterials and judiciously select biopolymers that elicit the most favorable biological responses. Strategic approaches such as targeted chemical modifications, precise control of gelation kinetics, and the development of composite materials serve as powerful methodologies for modulating and optimizing the inflammatory response elicited by hydrogels.^17^ The mechanical properties and chemical cues of GGMa hydrogels directly influence the macrophage behavior. Softer hydrogels have been shown to promote proinflammatory macrophages (M1), while stiffer hydrogels can shift macrophages toward the M2 phenotype. The GG-based hydrogels offer advantages such as printability and injectability and also a long-term resistance in physiological conditions. However, despite their influence on macrophage polarization, GG hydrogels alone may not be sufficient to induce a strong anti-inflammatory response in the body. Therefore, composites offer an alternative to enhance the properties of hydrogels, improving cell adhesion and proliferation.^18^ Fibrin hydrogels, obtained via thrombin-mediated polymerization of fibrinogen, represent an ECM-derived component that plays a key role in the wound-healing process guiding macrophage dynamics regulation.^19^ Combining the GG with the fibrin-based hydrogel may create a new biomaterial tunable for wound healing process, printability, and injectability. Because fibrinogen is enzymatically converted in situ, the biological and immunomodulatory effects observed are attributed to the fibrin network, rather than soluble fibrinogen. Unlike previously reported GelMA–fibrin or fibrin-only hydrogels, which primarily aim to enhance angiogenesis or mechanical strength, our GGMa–fibrin composite introduces a dual-network structure that integrates the ionic-covalent cross-linking of GGMa with the bioactive fibrillar architecture of fibrin.^20^ This configuration enables simultaneous control of mechanical stiffness and immunomodulatory signaling, representing a distinctive and previously unexplored approach for macrophage-guided tissue repair.

We engineered and systematically evaluated composite hydrogels comprising GMa, unmodified gellan gum (GG), and fibrin (Fib) to elucidate the influence of their mechanical properties on Raw 264.7 cells and primary murine macrophages. This study culminated in the development of a hydrogel platform with significant promise for advanced tissue regeneration and wound healing applications.

## Results and Discussion

### Mechanical and Surface Properties of the Hydrogel

Figures 1A–C show, respectively, the compressive modulus, the stress, and strain at rupture of hybrid hydrogels composed of unmodified gellan gum (5 mg/mL) and gellan gum methacryloyl (mg/mL): fibrin (mg/mL) ratios of 17:3 (GG_5_GMa_17_F_3_), 15:5 (GG_5_GMa_15_F_5_), 13:7 (GG_5_GMa_13_F_7_), and 11:9 (GG_5_GMa_11_F_9_). The compressive modulus of all hydrogel’s samples was statistically similar (*P* <0.05), from 3.00 ± 0.72 kPa to 4.58 ± 0.51 kPa. However, the gellan gum-based hydrogels broke in many fragments that generate errors in a compressive assay, probably due the heterogeneity of the samples; thus, we decided to calculate other mechanical properties that better fit with the hydrogel samples like the stress at rupture and the strain at the rupture. Stress at rupture is related to gel hardness, and strain at rupture reflects gel deformability.^21^ The strain at rupture values did not show differences between the samples. Thus, although the gellan gum and fibrin hybrid hydrogel had similar deformability at rupture, the hydrogel composition significantly affected their hardness. Hybrid hydrogels obtained in the ratio of 13:7 (GG_5_GMa_13_F_7_) were stiffer than the others, while those produced in the ratio of 17:3 (GG_5_GMa_17_F_3_) were softer. Hydrogels obtained in the ratios of 15:5 (GG_5_GMa_15_F_5_) and 11:9 (GG_5_GMa_11_F_9_) presented similar strains at rupture values. These results show that in the hybrid hydrogels GG_5_GMa_13_F_7_, the biopolymer chains were able to interact better to favor the structure of the three-dimensional network formed after the gelation processes. The fibrin changes the unmodified gellan gum and the gellan gum methacryloyl interaction, changing the hydrogel porosity and consequently the mechanical resistance.

**Figure 1:**
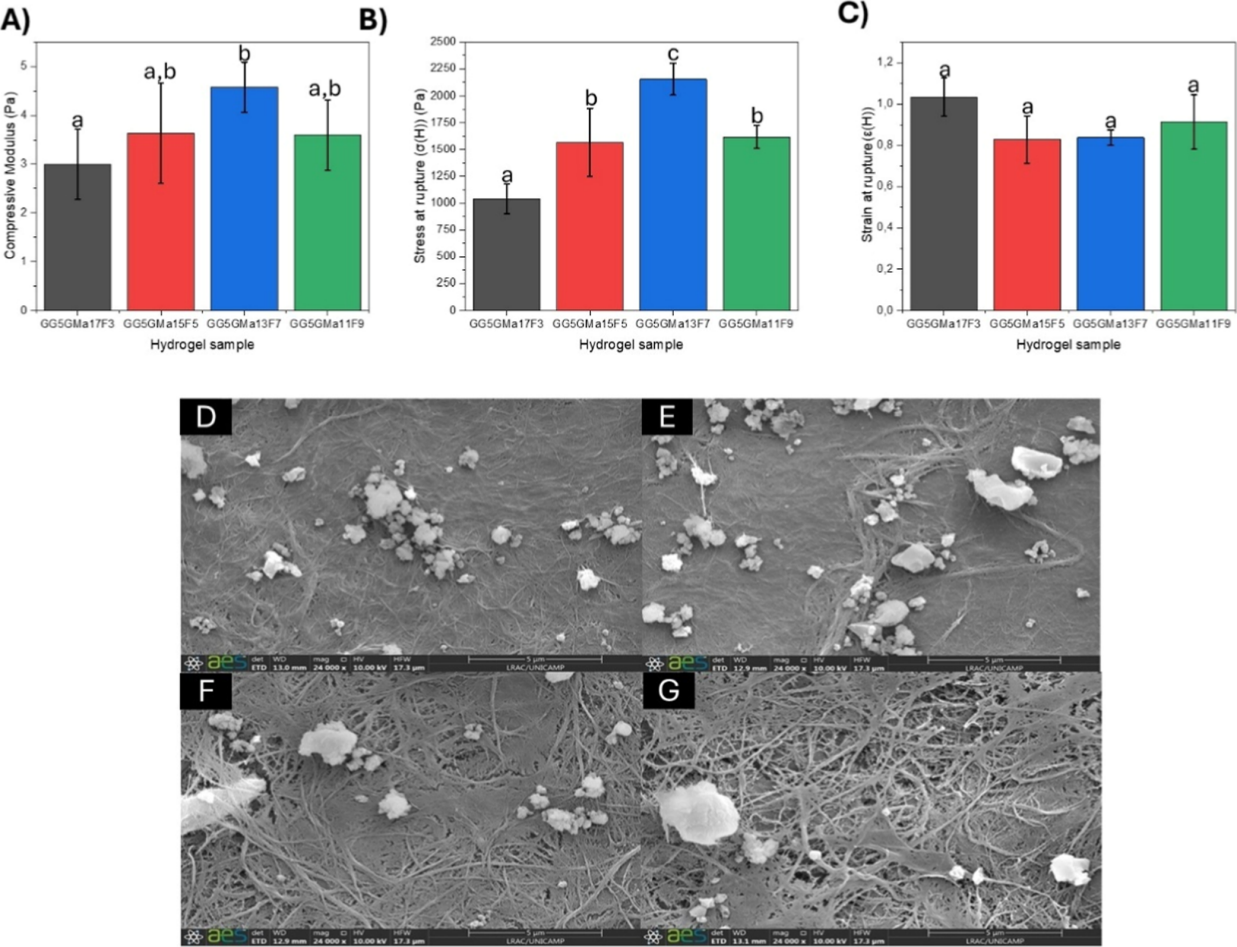
Characterization of the hydrogel construct. (A) Compressive modulus; (B) stress at rupture of hydrogel composites; (C) strain at rupture of hydrogel composites. Letters indicate differences between samples (*p* <0.05) by Tukey’s test. Scanning electron microscopy images of hydrogel composites: (D) GG_5_GMa_17_F_3_; (E) GG_5_GMa_15_F_5_; (F) GG_5_GMa_13_F_7_; and (G) GG_5_GMa_11_F_9_.

The microscopy of the hybrid hydrogels presented in Figure 1D–G provides a better understanding of the previously mentioned results. It is visible that at higher concentrations of gellan gum methacryloyl (GG_5_GMa_17_F_3_ and GG_5_GMa_15_F_5_ samples), the polymer matrix is more compact (Figure 1D and E). As the fibrin content increases, it becomes more porous (GG_5_GMa_13_F_7_ and GG_5_GMa_11_F_9_ samples), and the hydrogel microarchitecture shows more fiber structures and void pores (Figure 1F and G). A fully compacted network, as observed in the 17:3 ratio, generates a brittle hydrogel (weak under stress). In contrast, in the 13:7 ratio, there are compact and porous regions, which may have favored the development of stiffer gels (highest stress at rupture). The GGMa hydrogels have modulable stiffness due to variations in polymer concentration and a cross-linking mechanism. Xu et al. (2018)^22^ showed that the compressive modulus of GGMa hydrogels can range from 6.4 to 17.2 kPa, and the hydrogel stiffness was modified by the thiol-ene group ratio and calcium presence. Furthermore, the hydrogel composites have different stiffnesses due to the interaction between all of the polymer chains. Shin et al. (2012)^8^ combined gellan gum methacryloyl and methacrylated gelatin (GelMa) and found that the polymer concentration ratio affected the hydrogel stiffness; keeping the GelMa constant and ranging the GGMa concentration, the failure at the rupture significantly increased from 5.2 to 8.2.

### Proliferation and Viability of the Raw 264.7 Cells and Murine Macrophage on the Hydrogel Composite Surface

Gellan gum methacryloyl-based hydrogels have been extensively studied for tissue engineering applications and 3D cell culture, given the biocompatibility of these hydrogels.^22,23^ Modulating the composition of gellan gum methacryloyl-based hydrogels generates biomechanical and biochemical signals that promote cell proliferation and differentiation.^10^ In this study, we investigate the impact of GGMa hydrogels on two distinct cell lines: the immortalized Raw 264.7 cell line and murine-derived macrophage cells. To evaluate the cells’ response to the hydrogel microenvironment, we assessed cell behavior in 2D culture on the hydrogel surface and in 3D cell culture through encapsulation within the hydrogels.

First, we investigate whether the cells can adhere to and grow on the surface of the hydrogels and the effects of the presence of fibrinogen on the hydrogel composition. The Raw 264.7 cells grew and spread in all hydrogel compositions, resulting in an increase in the cell number after 5 days of culturing (Figure 2A). Furthermore, the fibrinogen concentration stimulated cell growth; the number of Raw 264.7 cells on the fifth day was higher in the GG_5_GMa_11_F_9_ and GG_5_GMa_13_F_7_ hydrogels compared to those in the GG_5_GMa_15_F_5_ and GG_5_GMa_17_F_3_ hydrogels (Figure 1B). The cell count for GG_5_GMa_11_F_9_ hydrogels was statistically different from those of GG_5_GMa_17_F_3_ and GG_5_GMa_11_F_5_ (*p* <0.05). However, despite the differences in the number of cells, regardless of the hydrogels’ composition, the viability of Raw 264.7 was above 90%, with no significant differences observed in hydrogel composition (Figure 2C, *p* <0.05). The fibrinogen concentration also affected the primary cell growth. The number of cells decreased after 5 days in cells cultured on the hydrogel with a lower fibrinogen concentration (GG_5_GMa_17_F_3_). In contrast, for the other 3 hydrogel compositions, the number of cells decreased from day 1 to day 3 and then increased from day 3 to day 5 (Figure 3D). On the fifth day, the number of cells on GG_5_GMa_17_F_3_ was lower than that on GG_5_GMa_15_F_5_, GG_5_GMa_13_F_7_, and GG_5_GMa_11_F_9_ (Figure 3E, *p* <0.05). The cell viability was significantly similar for all hydrogel samples during the first 2 days, hovering around 80% (Figure 2F, *p* <0.05). However, the viability of the cells on the GG_5_GMa_17_F_3_ hydrogel surface decreased on the third and fifth days (Figure 2F). In contrast, the other 3 compositions with higher fibrinogen concentrations maintained the cell viability around 80%.

**Figure 2:**
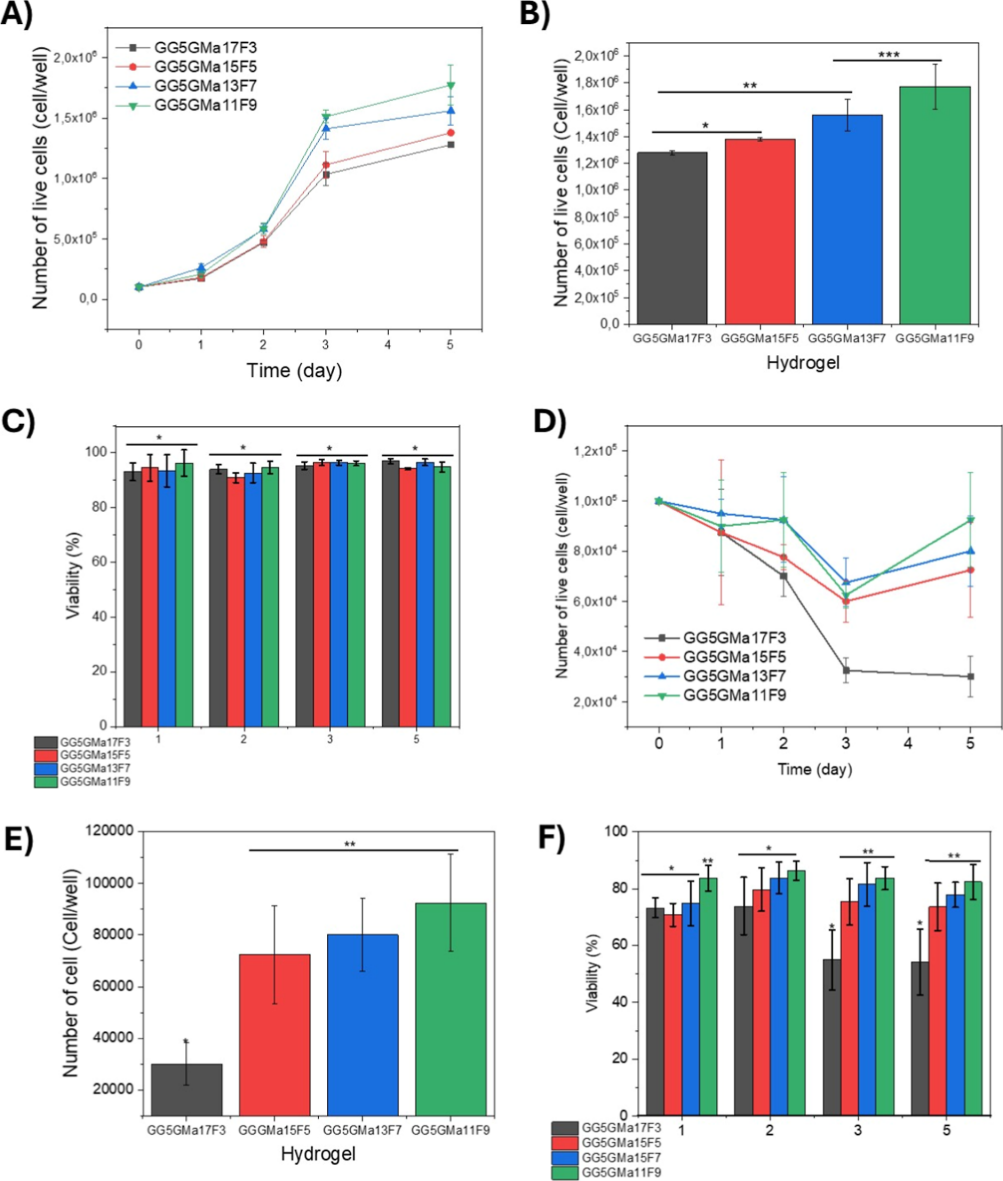
Quantification of the number of live cells and viability of the Raw 264.7 and murine-derived macrophages cultured on the hydrogel composite surface (2D surface). (A) Curve of the number of live Raw 264.7 cells through time. (B) Number of live Raw 264.7 cells on the fifth day of culture (*p* <0.05). (C) Raw 264.7 cell viability through time of culture (*p* <0.05). (D) Curve of the number of live murine-derived macrophage cells through time. (E) Number of live murine-derived macrophage cells on the fifth day of culture (*p* <0.05). (F) Murine-derived macrophage cells viability through time of culture (*p* <0.05).

**Figure 3:**
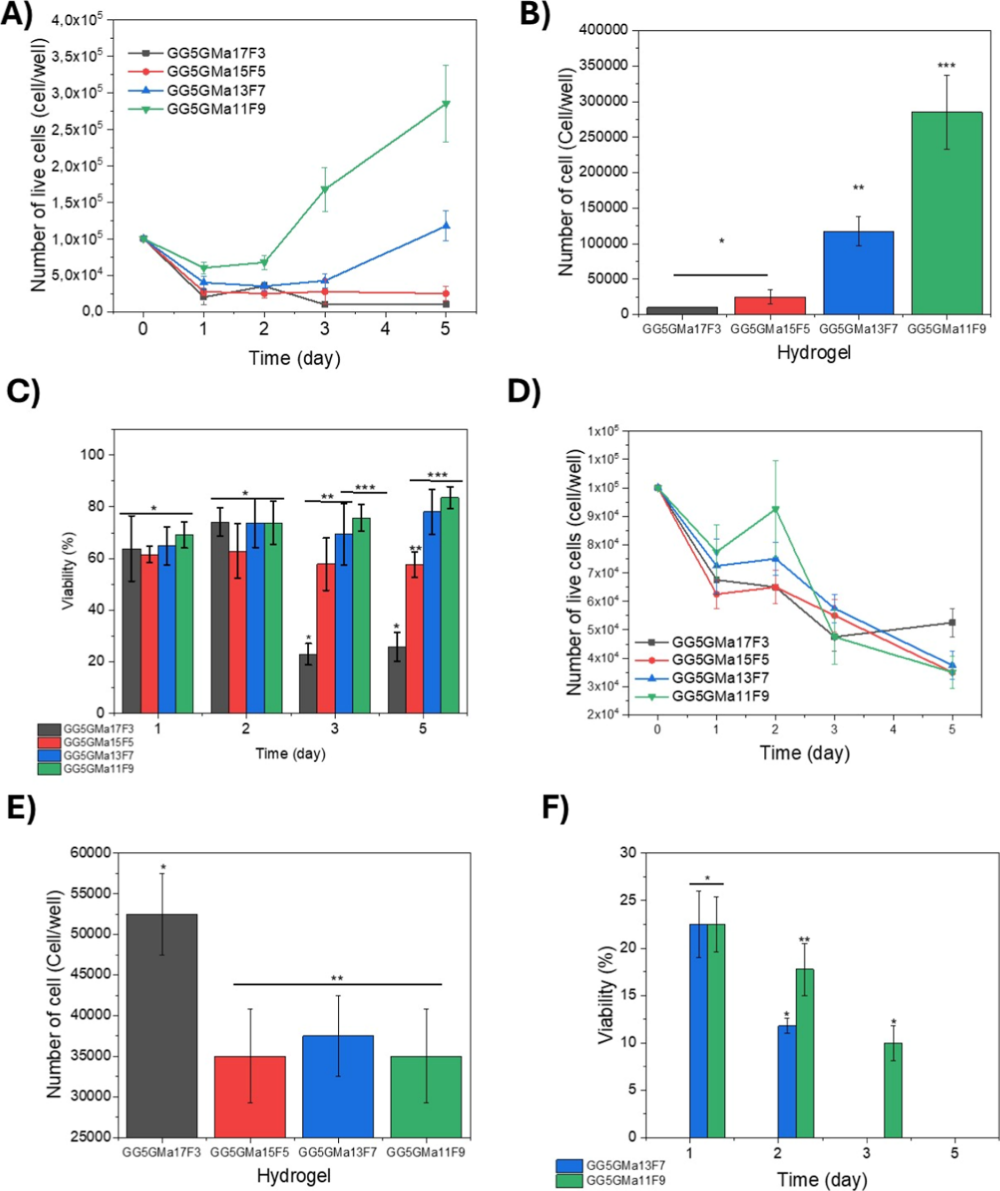
Quantification of the number of live cells and viability of the Raw 264.7 and murine-derived macrophage cultured encapsulated in hydrogel composites (3D culture). (A) Curve of the number of live Raw 264.7 cells through time. (B) Number of live Raw 264.7 cells on the fifth day of culture (*p* <0.05). (C) Raw 264.7 cell viability through time of culture (*p* <0.05). (D) Curve of the number of live murine-derived macrophage cells through time. (E) Number of live murine-derived macrophage cells on the fifth day of culture (*p* <0.05). (F) Murine-derived macrophage cells viability through time of culture (*p* <0.05).

The difference in the number of cell growth between the Raw 264.7 and the murine-derived cells can be explained by the primary cells’ sensitivity to the hydrogel surface.^24,25^ Although various hydrogel-based scaffolds can support macrophage culture, the chemical nature and mechanical properties directly affect cell viability. Generally, hydrogels inhibit the rate of cell proliferation, as demonstrated in the work of Li and Bratlie (2019);^18^ the authors quantified the macrophages M(0), M­(LPS), and M­(IL-4) cultured on the GGMa hydrogel surface, normalizing the cell number against those that grew on a flat glass surface. The GGMa hydrogels with thiol groups exhibited the lowest cell proliferation, ranging from 12 ± 1% to 21 ± 2%.

### Proliferation and Viability of the Raw 264.7 Cells and Murine-Derived Macrophage Encapsulated in the Hydrogel Composite

Herein, we also investigate the capacity of the hydrogel composites to encapsulate and sustain culture of murine-derived macrophage cells and Raw 264.7. Over time, the encapsulated Raw 264.7 lost viable cell numbers for the hydrogels GG_5_GMa_17_F_3_, GG_5_GMa_15_F_5_, and GG_5_GMa_13_F_7_ (Figure 3A). On the fifth day, the decrease in cell numbers was significantly lower for the hydrogels with lower fibrinogen concentrations, i.e., GG_5_GMa_17_F_3_ and GG_5_GMa_15_F_5_ (Figure 3B, *p* <0.05). Conversely, the hydrogels GG_5_GMa_13_F_7_ and GG_5_GMa_11_F_9_ (which have higher concentrations of fibrinogen) allowed the cells to proliferate. The cells in hydrogels GG_5_GMa_17_F_3_ and GG_5_GMa_15_F_5_ lost viability over time, whereas the cells in hydrogels GG_5_GMa_13_F_7_ and GG_5_GMa_11_F_9_ maintained a constant viability around 80%. The primary cells did not grow when encapsulated in any of the hydrogel samples, and cell numbers decreased after 5 days of culture (Figure 3D). On the fifth day, the cell number was lower for all conditions (Figure 3E). Nevertheless, the more porous hydrogels (GG_5_GMa_13_F_7_ and GG_5_GMa_11_F_9_) maintained high viability on the fifth day (Figure 3F, *p* <0.05), while all cells in the other gels died. The hydrogels GG_5_GMa_17_F_3_ and GG_5_GMa_15_F_5_ did not support the primary cells’ growth, as all cells died after 24 h of culturing. The cell viability in the hydrogel 7F was around 20% on the first day, decreased to approximately 10% on the third day, and all cells died on the fifth day. The hydrogel GG_5_GMa_11_F_9_ kept the cells viable over time, although the viability was low (around 10%) on the fifth day.

In hydrogel-based encapsulation systems, cell metabolism, phenotype, and morphology can change to adapt to a new 3D microenvironment. Additionally, the porosity of the hydrogels and their swelling capacity influence nutrient access for the cells, affecting the cell growth and proliferation. The hydrogel GG_5_GMa_11_F_9_ showed a higher number of cells because the presence of fibrinogen creates a fibrillar network with increased porosity, facilitating nutrient diffusion and cell mobility. Cells confined within a small space come into contact with biopolymers and solutions that differ significantly from their original environment, potentially leading to cell death via necrosis and triggering of the inflammatory process. Therefore, encapsulation systems based on hydrogels must create an appropriate microenvironment to sustain the metabolic activity and viability of encapsulated cells. The viability of macrophages inside the hydrogel is directly related to the stiffness and swelling of the hydrogel; the mechanical signals must support and guide the macrophages, while the swollen gel provides the necessary nutrients and oxygen for proliferation and viability.^26,27^

### Effect of 2D and 3D Culture on the Phenotype and Modulation of Primary Macrophages

The inflammatory response of the hydrogels was checked by primary macrophage differentiation into M1 and M2 types. Figure 4 shows fluorescence images of macrophages derived from murine bone marrow (primary cells) cultured for 72 h and stained with DAPI (blue) and F4/80 (red). The red color indicates the presence of the mouse F4/80 antigen, a 160 kDa glycoprotein expressed by murine macrophages. Although all primary cells express the mouse F4/80 antigen, control macrophages (cultured on glass) previously activated to M1 or M2 (Figure 4B and F) have more marked cell membrane edges than those not previously activated (Figure 4A and E). Furthermore, macrophages cultured on and inside the hybrid hydrogels also express the mouse F4/80 antigen, indicating that the macrophage phenotype was maintained even in contact with the polymer matrix composed of unmodified gellan (5 mg/mL), gellan gum methacryloyl (13 mg/mL), and fibrin (7 mg/mL) (Figure 4C, D, G, and H).

**Figure 4:**
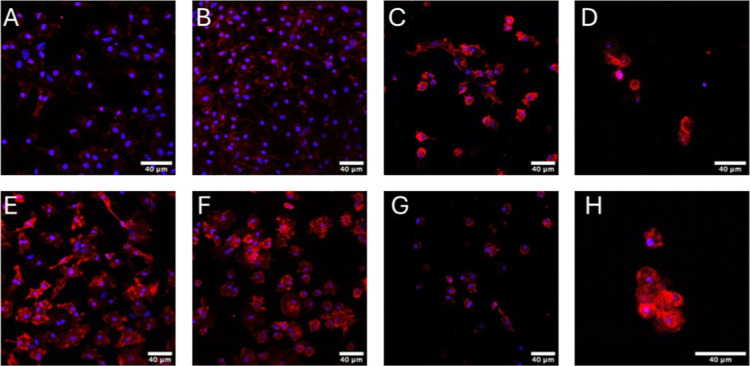
Fluorescence images of macrophages derived from murine bone marrow (primary cells) cultured for 72 h and stained DAPI (blue) and F4/80 (red). (A,E) Primary cells without previous polarization (macrophages control), (B,F) polarized previously for M1 macrophages (100 ng/mL LPS and 20 ng/mL IFN-γ) or M2 macrophages (20 ng/mL IL-4), respectively, (C,G) cultured on the surface of the hydrogel and (D,H) encapsulated macrophage in hydrogels.

Figure 5 presents fluorescence images of macrophages derived from murine bone marrow (primary cells) cultured for 72 h and stained with DAPI (blue) and Arginase1 or iNOS (green). Nonactivated (Figure 5A and E) and activated macrophages cultured on glass expressed inducible nitric oxide synthase (iNOS; Figure 5B) and Arginase1 protein (Figure 5F), established proinflammatory (M1) and prohealing (M2) macrophage phenotype markers, respectively. However, there is a slight increase in the fluorescence intensity of both markers in polarization-induced cells. Although cells deposited on the hybrid hydrogels (2D culture) also express Arginase1 and iNOS, (Figure 5C,G,H), the green intensity was considerably reduced, probably due to cell temporary phenotype differentiation into another macrophage type.^25^

**Figure 5:**
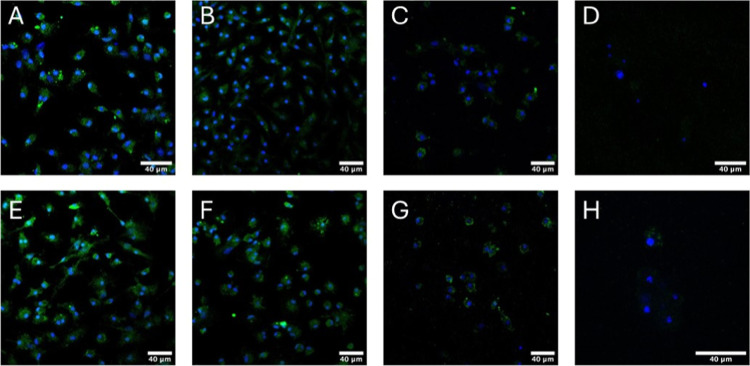
Fluorescence images of macrophages derived from murine bone marrow (primary cells) cultured for 72 h and stained DAPI (blue) and iNOS or Arginase1 (green). (A–D) Primary cells stained for DAPI + iNOS or (E–H) DAPI + Arginase1 without previous polarization (macrophage control); (A,E), polarized previously for M1 macrophages (100 ng/mL LPS and 20 ng/mL IFN-γ); (B) or M2 macrophages (20 ng/mL IL-4); (F), cultured on the gel (2D culture); (C,G) cultured on the surface of the hydrogel; and (D,H) encapsulated macrophage in hydrogels.

Previous work has demonstrated that fibrin and fibrinogen activate opposing macrophage functions: fibrin is anti-inflammatory, while fibrinogen is inflammatory.^19^ Besides, RAW 264.7 macrophages cultivated on gellan gum methacryloyl gels had their phenotype modulated by the mechanical and chemical properties of the hydrogels. In general, stiffer matrices modulated the M1 phenotype toward M2-like characteristics.^28^ Li and Bratlie (2018)^18^ demonstrated the correlation between the mechanical properties of gellan gum hydrogels and the macrophage activation; stiffer gellan gum hydrogels induced lower nitrite production and elevated urea production, modulating the M1 phenotype to M2 phenotype. On the other hand, softer hydrogels had less urea production and arginase activity.^18^

It is important to note that the present immunocytochemical analysis provides qualitative insights into macrophage modulation within the hydrogel matrix. Due to the limited number of viable cells recovered from the 3D hydrogels (typically below 20% after 5 days of culture), quantitative approaches such as qPCR, ELISA, or flow cytometry were not feasible. Flow cytometry, in particular, requires a large population of dissociated and viable cells to reliably distinguish macrophage subpopulations (M1, M2a, M2c, etc.). Although quantitative analysis is highly valuable for this type of study, the qualitative assessment performed here was sufficient to support the objectives of this work, especially considering the experimental limitations described above.

## Conclusion

This study demonstrated the potential of gellan gum methacryloyl-based composite hydrogels as versatile scaffolds for macrophage culture and immunomodulation. By fine-tuning the composition of GGMa, gellan gum, and fibrinogen, we were able to modulate the mechanical properties of the hydrogels that directly impact the macrophages’ viability, proliferation, and differentiation. Despite that, the low viability of the primary macrophage encapsulated is a consequence of low nutrient diffusion and mechanical pressure in the cells. Changing the hydrogels’ porosity is an alternative to improving the cell viability over time, such as creating microporous annealed particle (MAP) scaffolds using droplet-microfluidics or extrusion methods. Furthermore, the degree of modification of the hydrogel also affects its mechanical properties and biological behavior; therefore, further investigation on different propositions of gellan gum and methacrylic acid is a gap that may be investigated. Although, the hydrogels maintain the M1 macrophages and a few expressions of M2 macrophages, highlighting the potential of this material for wound healing applications.

## Material and Methods

### Material

Gellan gum (GelzanTM CM Gelrite), methacrylate anhydride, 2-hydroxy-4′-(2-hydroxyethoxy)-2-methylpropiophenone (I 2959), fibrinogen from bovine plasma, and Raw 264.7 cells (murine macrophage cells) were obtained from Sigma-Aldrich (St. Louis, USA). The other reagents were of analytical grade.

### Synthesis of Methacryloyl Gellan Gum (GMa)

Methacrylate gellan gum was synthesized by substituting the hydroxyl groups in the repeating units of gellan gum with methacrylate anhydride.^9^ Briefly, 1 g of gellan gum was dissolved in 100 mL of deionized water in a round-bottom flask and heated to 90 °C for 30 min under constant stirring. Next, the mixture was slowly cooled to 50 °C, and 4 mL of methacrylate anhydride was added to the gellan gum. The pH was maintained at 8 using 5 M NaOH. After 4 h, the product was dialyzed (cellulose membrane, molecular weight cutoff 12–14 kDa, Sigma-Aldrich) against deionized water for 5 days, with the deionized water being refreshed twice daily. The final product was lyophilized and stored at −20 °C.

### Preparation of GG and GMa Dispersion

Methacrylated gellan gum (3.7% w/w) was mixed with the photoinitiator I-2959 (0.5% w/w) and dissolved in deionized water while stirring at 70 °C for 2 h. Unmodified gel gum was dissolved to a concentration of 1.75% (w/w) in deionized water, also stirring at 70 °C for 2 h. The dispersions of modified and unmodified gel gum were sterilized by passing them through a 0.45 μm filter, followed by a 0.22 μm filter, for a 15 mL conical tube in a sterile laminar flow hood.

### Preparation of Fibrinogen

240 mg of fibrinogen was added from a 60 mm Petri dish containing 3 mL of Tris-buffered saline solution (TBS). Four L of TBS (4 L) was prepared with 17.44 g of Tris HCl, 2.56 g of Tris base, 32 g of NaCl, and 0.8 g of KCl. After 5 min, the plates were sealed and incubated at 37 °C for 4 h to allow fibrinogen to dissolve completely. Then, the fibrinogen was pipetted into dialysis tubes (cellulose membrane, molecular weight cutoff 12–14 kDa, Sigma-Aldrich). The fibrinogen was dialyzed against TBS buffer with gentle agitation for 24 h; after 12 h, the dialysis solution was replaced. After the subsequent 12 h, the fibrinogen solution was removed from the dialysis tube, transferred to a 15 mL conical tube, and sterilized by passing it through a 0.22 μm filter. The BCA kit determined the final fibrinogen concentration (Protein Assays, Thermo Fisher Scientific). The final product was stored at −80 °C.

### Preparation of the GGMa, GG, Fib Hydrogel Composite

The previously prepared dispersions of unmodified gellan gum (GG), methacryloyl gellan gum (GMa), and fibrinogen (Fg) were mixed to obtain different compositions of hydrogels, as shown in Table 1. Subsequently, the enzyme thrombin was added to the mixtures in the proportion of 90:10 (fibrinogen:thrombin) to form fibrin fibers. Consequently, in all experiments, the resulting hydrogel component is fibrin, and references to “fibrinogen” in formulations denote its precursor state before thrombin addition”.

**Table 1:** Description of the Different Compositions of the Hydrogels and Their Nomenclature

hydrogel composition	GG unmodified gellan gum % w/v	GMa methacryloyl gellan gum % w/v	Fg fibrinogen % w/v
GG_5_GMa_17_F_3_	5	17	3
GG_5_GMa_15_F_5_	5	15	5
GG_5_GMa_13_F_7_	5	13	7
GG_5_GMa_11_F_9_	5	11	9

### Mechanical Characteristics of the Hydrogel

100 μL of the hydrogel mixtures was deposited in cylindrical molds (0.5 cm) made of laminated polydimethylsiloxane (PDMS) that were previously laser-cut. The systems were exposed to UV radiation (365 nm, 7 mW/cm^2^) for 3 min. The hydrogels were removed from the cylindrical molds and underwent mechanical and rheological testing. The uniaxial compression analysis using a TA-XT Plus Texture Analyzer (Stable Micro Systems, UK) aimed to evaluate the mechanical properties of the hydrogels. An acrylic cylindrical plate geometry with a 40 mm diameter, lubricated with low viscosity silicone oil, was employed to compress the construct at a rate of 0.5 mm/s to 80% of its initial height. All measurements were conducted at 25 °C in triplicate. Each hydrogel was compressed during the test, and the maximum force value was recorded for each measurement. Hencky stress (σH) and strain (εH) at rupture were calculated from the peak maximum force according to eqs 1 and 2, respectively.

1 $$\sigma_{H}=F_{\left(t\right)}\frac{H_{\left(t\right)}}{H_{\left(o\right)}\cdot A_{\left(o\right)}}$$

2 $$\varepsilon_{H}=\ln\left(\frac{H_{\left(t\right)}}{H_{\left(o\right)}}\right)$$

where *F*_(*t*)_ is the force at time *t*, *A*_(o)_ and *H*_(o)_ are the initial area and height of the sample, respectively, and *H*_(*t*)_ is the height at time *t*.

### Scanning Electron Microscopy (SEM) of the Hydrogel

The hydrogel morphology surfaces were characterized by using scanning electron microscopy. For that, 100 μL of the hydrogel mixtures was deposited onto coverslips (=13 mm, Perfecta) inside 24-well plates. The plates were exposed to UV light (365 nm, 7 mW/cm^2^) for 3 min before being added to the culture medium. The hydrogels were washed twice with phosphate-buffered saline (PBS). All samples were then prepared according to the following procedure: (i) fixation with 4% PFA, (ii) dehydration using a series of ethanol concentrations (30, 50, 70, 95, and 100% volume of ethanol mixed with deionized water), and finally, (iii) chemical “critical point drying” using hexamethyldisilane (HMDS) (Sigma-Aldrich). The samples on coverslips were removed from the plates and fixed onto stubs with carbon tape. The coverslips were attached to SEM stubs using carbon tape and sputter-coated with gold in a Sputter Coater EMITECH instrument (model K450, Kent, United Kingdom). Images were obtained using scanning electron microscopy (SEM) (LEO Electron Microscopy/Oxford, model 440i, Cambridge, England).

### Immortalized Macrophage Cell Culture

Raw 264.7 cells were cultured in complete Dulbecco’s modified Eagle’s medium (DMEM) supplemented with 10% bovine calf serum (BCS), 100 U/L penicillin, and 100 μg/L streptomycin at 37 °C in 5% CO_2_. The medium was replaced every 2 days until the desired confluence was achieved.

### Murine Macrophage Cell Culture

In order to obtain macrophages derived from murine bone marrow (primary cells), Balb/c mice aged 10 weeks were euthanized using intraperitoneal injectable anesthetics consisting of a combination of xylazine (Anasedan, 300 mg/kg, Sespo Industria e Comércio Ltd., Brazil) and ketamine (Dopalen, 30 mg/kg, Sespo Industria e Comércio Ltda., Brazil). After confirming the animal’s loss of consciousness, the cervical dislocation method was applied. Under aseptic conditions, a skin incision was made in the anterior region of both thighs, severing the muscle layer to expose the femurs. After its exposure, the femurs between the femur-iliac and femur-tibial joints were sectioned. Excess leg muscle was removed by holding the bone’s end with forceps and scissors. Subsequently, the leg bones proximal to each joint were carefully cut using sharp scissors soaked in ethanol. Cells were extracted by washing the bone marrow of the femur and tibia with phosphate buffered saline solution (PBS) containing fetal bovine serum (FBS) and 100× penicillin/streptomycin. Subsequently, 4 × 10^6^ cells were seeded in low-adherence culture dishes and maintained in high-glucose DMEM containing 20% L929 cell supernatant (source of macrophage colony-stimulating factor), 20% FBS, 0.4 mM of sodium pyruvate, and 0.2 mM of β-mercaptoethanol. Cells were kept in an incubator at 37 °C with 5% w/v CO_2_.

### Flat Cell Culture on the Hydrogel Surface

The scaffold was prepared with 100 μL of the dispersions containing the hydrogel mixtures and seeded on a glass coverslip (ϕ = 13 mm, Perfecta) inside 24-well plates. The plates were exposed to UV light (365 nm, 7 mW/cm^2^) for 3 min. The Raw 264.7 and primary cells were lifted and seeded at a final concentration of 1 × 10^5^ cells/well on each hydrogel.

### Cell Encapsulation in the Hydrogel

Cells at the final concentration of 1 × 10^5^ cells/well were mixed with the fibrinogen solution before preparing the hydrogel mixture. 100 μL of the hydrogels with cells was deposited on coverslips (ϕ = 13 mm, Perfecta) inside 24-well plates. The plates were exposed to UV light (365 nm, 7 mW/cm^2^) for 3 min.

### Cell Viability

On days 1, 2, 3, and 5, the hybrid hydrogels were evaluated for cell concentration and viability. Initially, the medium was removed from the well and placed in a 15 mL conical tube. Subsequently, 1.0 mL of PBS-EDTA was used to break up the hybrid hydrogels, releasing the cells from the polymer matrix. After complete release, PBS–EDTA and cells were dispensed into the same 15 mL conical tube that held the medium. Following gentle mixing, 10 μL of this dispersion was mixed with 10 μL of trypan blue dye. Finally, the cells were counted through direct observation under a microscope in a Neubauer chamber. In this context, cell viability was assessed by the cell density over time.

### Immunocytochemistry

Primary cell cultures on hydrogel composite surfaces encapsulated into the hydrogels were prepared as previously described. Moreover, cells (1 × 10^5^ cells/well) were deposited on the coverslips (ϕ = 13 mm) inside 24-well plates to be used as controls. As controls of macrophage activation, 100 ng/mL LPS and 20 ng/mL IFN-γ to induce M1 macrophage activation and 20 ng/mL IL-4 to induce M2 macrophage activation were used. After 72 h of induced activation, all samples (hydrogels and controls) were prepared and analyzed as described by Mehrban et al.^29^

### Statistical Analysis

All individual experiments were performed in triplicate. All statistical analyses were performed using Minitab 18 software. The one-way analysis of variance (ANOVA) with a post hoc Tukey’s test was done. Differences between conditions were considered significant at *p* <0.05 (*).
